# Correction to: Myocardin regulates mitochondrial calcium homeostasis and prevents permeability transition

**DOI:** 10.1038/s41418-019-0436-0

**Published:** 2019-10-22

**Authors:** Wajihah Mughal, Matthew Martens, Jared Field, Donald Chapman, Jianhe Huang, Sunil Rattan, Yan Hai, Kyle G. Cheung, Stephanie Kereliuk, Adrian R. West, Laura K. Cole, Grant M. Hatch, William Diehl-Jones, Richard Keijzer, Vernon W. Dolinsky, Ian M. Dixon, Michael S. Parmacek, Joseph W. Gordon

**Affiliations:** 1 Department of Human Anatomy and Cell Science, University of Manitoba, Winnipeg, MB Canada; 2 Diabetes Research Envisioned and Accomplished in Manitoba (DREAM) Theme, Children’s Hospital Research Institute of Manitoba, Winnipeg, MB Canada; 3 Department of Biological Science, University of Manitoba, Winnipeg, MB Canada; 4 Department of Medicine, Penn Cardiovascular Institute, Hospital of the University of Pennsylvania, Philadelphia, PA USA; 5 Department of Physiology and Pathophysiology, University of Manitoba, Winnipeg, MB Canada; 6 Institute of Cardiovascular Sciences, St. Boniface Research Centre, Winnipeg, MB Canada; 7 College of Nursing, University of Manitoba, Winnipeg, MB Canada; 8 Department of Pharmacology and Therapeutics, University of Manitoba, Winnipeg, MB Canada; 9 The Biology of Breathing Theme, Children’s Hospital Research Institute of Manitoba, Winnipeg, MB Canada; 10 Faculty of Health Disciplines, Athabasca University, Edmonton, MB Canada; 11 Department of Surgery, University of Manitoba, Winnipeg, MB Canada

**Keywords:** Cell biology, Molecular biology

**Correction to:Cell Death & Differentiation**

10.1038/s41418-018-0073-z

The original PDF version of this article incorrectly showed the copyright holder to be ‘ADMC Associazione Differenziamento e Morte Cellulare 2018’, when the correct copyright holder is ‘The Authors 2018’. This has been corrected in the PDF version of the article. The HTML version was correct from the time of publication.

